# 2-Deoxy-D-glucose Alleviates Cancer Cachexia-Induced Muscle Wasting by Enhancing Ketone Metabolism and Inhibiting the Cori Cycle

**DOI:** 10.3390/cells11192987

**Published:** 2022-09-25

**Authors:** Lulu Wei, Ranran Wang, Junaid Wazir, Kai Lin, Shiyu Song, Li Li, Wenyuan Pu, Chen Zhao, Yong Wang, Zhonglan Su, Hongwei Wang

**Affiliations:** 1 Medical School, State Key Laboratory of Analytical Chemistry for Life Science & Jiangsu Key Laboratory of Molecular Medicine, Nanjing University, Nanjing 210093, China; 2 Department of Dermatology, the First Affiliated Hospital of Nanjing Medical University, Nanjing 210029, China

**Keywords:** cancer cachexia, muscle wasting, glucose metabolism, Cori cycle, ketone

## Abstract

Cachexia is characterized by progressive weight loss accompanied by the loss of specific skeletal muscle and adipose tissue. Increased lactate production, either due to the Warburg effect from tumors or accelerated glycolysis effects from cachectic muscle, is the most dangerous factor for cancer cachexia. This study aimed to explore the efficiency of 2-deoxy-D-glucose (2-DG) in blocking Cori cycle activity and its therapeutic effect on cachexia-associated muscle wasting. A C26 adenocarcinoma xenograft model was used to study cancer cachectic metabolic derangements. Tumor-free lean mass, hindlimb muscle morphology, and fiber-type composition were measured after in vivo 2-DG administration. Activation of the ubiquitin-dependent proteasome pathway (UPS) and autophagic–lysosomal pathway (ALP) was further assessed. The cachectic skeletal muscles of tumor-bearing mice exhibited altered glucose and lipid metabolism, decreased carbohydrate utilization, and increased lipid *β*-oxidation. Significantly increased gluconeogenesis and decreased ketogenesis were observed in cachectic mouse livers. 2-DG significantly ameliorated cancer cachexia-associated muscle wasting and decreased cachectic-associated lean mass levels and fiber cross-sectional areas. 2-DG inhibited protein degradation-associated UPS and ALP, increased ketogenesis in the liver, and promoted ketone metabolism in skeletal muscle, thus enhancing mitochondrial bioenergetic capacity. 2-DG effectively prevents muscle wasting by increasing ATP synthesis efficiency via the ketone metabolic pathway and blocking the abnormal Cori cycle.

## 1. Introduction

Cachexia is a multiorgan syndrome characterized by progressive body weight loss and associated with a wide range of acute and chronic diseases, such as cancer, cardiovascular disease, infection, and chronic inflammatory conditions [1,2]. Cachexia is particularly prevalent in cancer patients, affecting 50–80% of patients and causing approximately 20% of cancer deaths [3]. The appearance of cachectic syndrome in cancer patients negatively affects quality of life and causes reduced tolerance and responsiveness to chemotherapy, which is associated with a worsening prognosis and higher mortality rate [4]. Skeletal muscle wasting is the central event of cancer cachexia, which results from an imbalance between protein synthesis and degradation. There is no evidence that cancer cachexia patients have suppressed myofibrillar protein synthesis based on studies on muscle protein turnover kinetics [5,6]. Therefore, enhanced protein degradation is likely the major cause for the induction of muscle loss, in which the increased activation of the ubiquitin-dependent proteasome pathway (UPS) and the autophagic–lysosomal pathway (ALP) plays a crucial role [7,8]. The UPS is the main protein degradation system in eukaryotic cells [9], and by activating the UPS in skeletal muscle, structural and contractile proteins are degraded, leading to atrophy and reduced muscle function [10]. ALP is a major intracellular proteolytic system that removes long-lived proteins and organelles in mammalian cells [11,12]. ALP activation can be seen during the early stages of cachectic weight loss [13,14,15], and in cancer patients, muscle lysosomal enzyme activity has been linked to weight loss [16]. Cancer cachexia-associated muscle atrophy results from an imbalance of energy requirements and energy uptake [17]. Patients with cancer cachexia manifest both malnutrition and metabolic stress, in which increased energy expenditure, insulin resistance, high plasma glucose levels, and excess catabolism are the typical metabolic changes [18]. The increases in futile cycles, such as the Cori cycle, directly contribute to elevated resting energy expenditure in cancer cachexia. Since cancer cells demonstrate an increase in glucose uptake but cannot effectively use glucose due to the Warburg effect [19], lactate derived from tumors and other organs is converted into hepatic glucose via gluconeogenesis to maintain the glucose supply and reduce the plasma lactate level. Glucose production and energy expenditure are enhanced by the Cori cycle between organs and cancerous cells, and blocking the futile Cori cycle could be a viable treatment option for cancer cachexia. Another important feature of cancer cachexia is the loss of adipose tissue; although the underlying mechanism is largely unknown, increased adipocyte lipolysis has been attributed to this process. The breakdown of triacylglycerol causes the release of free fatty acids (FFAs); therefore, elevated FFA levels are commonly observed in patients with cancer cachexia [20]. The increased plasma FFA could not only be used as a source for energy generation, but could also be converted into ketone bodies and released from the liver as another form of an energy source. As the mitochondria of cancer cells are largely dysfunctional, they are unable to utilize fatty acids and ketone bodies, which healthy cells can use as energy sources [21]. Therefore, in cancer cachexia, ketone bodies might provide an alternative metabolic pathway in skeletal muscle, and its beneficial effects have been well described [22]. Although cancer cachexia patients manifest elevated plasma glucose levels, both carbohydrate utilization and amino acid incorporation are decreased in the muscles of cancer cachexia patients [23]. Therefore, we wondered whether replacing glucose with other energy generation sources would be beneficial to cancer cachexia-associated muscle wasting. 2-Deoxy-D-glucose (2-DG) is a glucose analog and is similar to glucose in structure [24]. Research shows that 2-DG suppresses glycolysis by competing with glucose to bind hexokinase, the first rate-limiting enzyme of glycolysis [25]. The therapeutic effects of 2-DG on multiple metabolism-associated disorders have been reported, including promoting neuron survival in models of Parkinson’s disease [26], improving renal function [27], and inhibiting the carcinogenic process in breast cancer [28]. However, the functional role of 2-DG application in cancer cachexia is still not known. The purpose of this study was to determine the therapeutic potential of 2-DG in cachectic skeletal muscle. We found that in a C26 tumor-bearing cachectic mouse model, cachectic skeletal muscle manifested an altered glucose metabolic pattern by switching oxidative phosphorylation into glycolysis; the 2-DG treatment promoted ketone body utilization in cachectic muscle, effectively mitigating muscle atrophy by inhibiting UPS and ALP pathway activation. Additionally, in the liver, the 2-DG treatment promoted ketogenesis but reduced gluconeogenesis and the Cori cycle. This discovery established a novel therapeutic strategy for improving patients’ quality of life and alleviating systemic symptoms associated with cancer cachexia.

## 2. Materials and Methods

### 2.1. Cell Culture

The mouse myoblast C2C12 was obtained from the American Type Culture Collection (ATCC, Manassas, VA, USA) and cultured in Dulbecco’s modified Eagle’s medium (DMEM), which was supplemented with 10% fetal bovine serum, penicillin (100 mg/mL), and streptomycin (100 mg/mL). To induce myotube formation, cells were grown to 100% confluency and switched to a DMEM containing 2% horse serum (Gibco Carlsbad, CA, USA) and 1% penicillin/streptomycin for up to 4–7 days. The mouse tumor cell line colorectal adenocarcinoma colon 26 (C26) was purchased from Cell Lines Service (CLS, Eppelheim, Germany) and maintained in RPMI 1640 medium with L-glutamine (Life Technology, New York, NY, USA), containing 10% fetal bovine serum, penicillin (100 mg/mL), and streptomycin (100 mg/mL). Cells were treated with 5 mM 2-DG (HY-13966, MedChemExpress (Monmouth Junction, NJ, USA)), 20 μM cisplatin (HY-17394, MedChemExpress (Monmouth Junction, NJ, USA)) or 25 μM chloroquine (HY-17589A, MedChemExpress (Monmouth Junction, NJ, USA)), 100 μM BHB (HY-113378, MedChemExpress (Monmouth Junction, NJ, USA)), 20 ng/mL TNF-*α* (10602-HNAE, Sino Biological Inc., Beijing, China), and 40 ng/mL IFN-*γ* (50709-M08H, Sino Biological Inc.) for 24 h. The C26-derived conditioned medium was aspirated at the end of the C26 culture for 48 h, filtered by a 0.22 μM filter, collected, and saved at 4 °C. All the cell lines were incubated at 37 °C in a humidified chamber with 5% CO_2_.

### 2.2. Cancer Cachexic Mouse Model

Six-week-old male BALB/c mice (obtained from the Model Animal Research Center of Nanjing University) were acclimated for one week under specific pathogen-free (SPF) conditions. Each mouse was kept in an individually ventilated cage (IVC) with free access to drinking water, a basal diet, and humidity under conditions of controlled humidity, light (12 h light/12 h dark cycle), and temperature. All animal experiments were carried out in accordance with protocols approved by Nanjing University’s Institutional Animal Care and Use Committee. A total of 30 mice were then randomly assigned to one of three groups: normal (NC; *n* = 10), tumor-bearing control (cachexia; *n* = 10) or cachexia plus 2-DG (cachexia+2-DG; *n* = 10). On day 0, colon 26 cells (5.0 × 105) in 0.1 mL phosphate-buffered saline were inoculated subcutaneously into the left flanks of Cachexia and Cachexia+2-DG mice on day 0. The Cachexia+2-DG group mice received an intraperitoneal injection of 0.5 g/kg 2-DG (dissolved in PBS, purchased from MCE, HY-13966, China) every day, starting at day 7 after tumor injection. The mice were sacrificed when they lost 20% of their body weight or reached a tumor volume of 1500 mm^3^. Tumors, muscles, and other organs were dissected rapidly and stored at −80 °C until further analyses or fixed with 4% formaldehyde for histological staining. Food intake was calculated by taking the remaining food weight each day and subtracting the current day’s food weight from the previous day’s weight. Cancer cachexic mouse model experiments were repeated three times to ensure reliable experimental repeatability.

### 2.3. Histological Analysis

Mice were sacrificed, and tissues were fixed in 4% formaldehyde overnight, followed by dehydration and embedding in paraffin. For histopathological evaluation, 2 μM-thick skeletal muscle sections were stained with hematoxylin and eosin (H&E) and periodic acid–Schiff (PAS) and were examined by light microscopy. Immunofluorescence staining of paraffin sections was performed using fluorochrome-conjugated antibodies, following the previously reported methods [29]. Masson trichrome staining was carried out with a Masson modified IMEB stain kit (K7298, IMEB Inc. San Marcos, CA, USA) in accordance with the protocol. Under an FV10i laser scanning confocal microscope (Olympus, Center Valley, PA, USA), the specimens were examined.

### 2.4. Immunofluorescence

Cells were plated in 12-well plates coated with poly-D-lysine (0.1 mg/mL) and induced to differentiate. After treatment, the cells were fixed in 4% paraformaldehyde for 30 min, permeabilized with 0.1% Triton X-100 in PBS, and incubated overnight at 4 °C with an anti-myosin heavy-chain (MHC) (#MAB4470, R&D Systems) (1:100) in 1% BSA/PBST. The cells were then incubated for 1 h at room temperature with a fluorescence-labeled secondary anti-mouse antibody (1:1000) and DAPI (1:1000). Under an FV10i laser scanning confocal microscope, samples were examined and images were captured (Olympus, Center Valley, PA, USA). The mean gray value was used to indicate the difference and calculated by IntDen (integrated density)/area via ImageJ.

### 2.5. ATP Level Detection

ATP levels were determined according to the manufacturer’s instructions using a firefly luciferase-based ATP assay kit (Beyotime, S0026, Shanghai, China). Based on the following formula, we calculated the relative ATP level: relative ATP level = ATP value/protein value.

### 2.6. Measurement of AcCoA

The PicoProbe AcCoA assay kit (ab87546, Abcam, Cambridge, UK) was used to assess the AcCoA content in total or cytosolic fractions, following the manufacturer’s protocol.

### 2.7. Lactate and Pyruvate Assay

The lactate content was quantified using a Lactic Acid LD kit (Keygen, KGT023, Nanjing, China), following the manufacturer’s protocol. The pyruvate content was measured using a pyruvate kit (A081, Nanjing Jiancheng Bioengineering Institute, Nanjing, China) in accordance with the manufacturer’s guidelines. The assays of lactate and pyruvate contents were detected using a spectrophotometer (Thermo) at 570 nm and 505 nm, respectively.

### 2.8. Ketone Body Assay

Ketone body content was determined using a Ketone Body Assay Kit (ab272541, Abcam, Cambridge, UK), following the manufacturer’s guidelines. The absorbance was detected using a spectrophotometer (Thermo) at 340 nm. The total ketone body (TKB) concentration was calculated as [TKB] = [acetoacetic acid (AcAc)] + [3-hydroxybutyric acid (BHB)]. 

### 2.9. Western Blot

Total cell lysates were solubilized in ice-cold RIPA lysis buffer (P0013B, Beyotime Biotechnology, Shanghai, China) containing protease and phosphatase inhibitor cocktails (MedChemExpress (Monmouth Junction, NJ, USA). Incubation with the primary antibodies was performed after blocking the membranes with 5% bovine albumin at room temperature for 2 h. Standard procedures were followed for the detection of the signals using the following antibodies: STAT3 (CST, #9145), phosphorylated STAT3 (CST, #9139), atrogin-1 (Satan Cruz, sc166806), MuRF-1 (Santa Cruz, sc398608), MHC (R&D Systems, #MAB4470), p62 (CST, #5114s), LC3 (Proteintech, 14600-1-AP), cleaved caspase3 (CST, #9664T), cleaved PARP (CST, #5625), phospho-P70 S6K (Beyotime, AF5899), phospho-EIF4EBP1 (Beyotime, AF5806), GLUT1/SLC2A1 rabbit mAb (Abclonal, A11727), Bdh1 rabbit pAb (Abclonal, A3763), HMGCS2 antibody (Affinity, DF14319), and GAPDH (Proteintech, 60004-1-Ig). Subsequently, at room temperature, the membranes were washed and incubated for 2 h with peroxidase-conjugated secondary antibodies (Bioworld). The enhanced chemiluminescence (ECL) system was used to record chemiluminescent images of immunostained bands on membranes on X-ray films, following the manufacturer’s protocol.

### 2.10. Real-Time PCR Assay

Total RNA was extracted from tissues and cell lines using the RNA isolator total RNA extraction reagent (R401-01, Vazyme, Nanjing, China), and reverse transcription was performed using the PrimeScript RT Master Mix (TaKaRa, Otsu, Japan) and subjected to an SYBR Green quantitative real-time PCR using the PCR Master Mix (Life Technologies, Austin, TX, USA), following the manufacturers’ instructions. Q-PCRs were performed on an ABI 7500 real-time PCR system (Applied Biosystems, Waltham, MA, USA). The expression levels were normalized to the actin levels. The relative expression was calculated using the 2 ∆∆Ct method. Detailed primer sequences are provided in Supplementary Table S1.

### 2.11. Forced Swimming Test

The forced swimming test was performed on all groups of mice 30 min after the last administration. In brief, the mice were placed in a 30 cm-deep acrylic plastic pool (40 × 40 × 40 cm) filled with fresh water (26 ± 1 °C). The swimming time was recorded after exhaustion was defined as the loss of coordinated movements and failure to rise to the surface within 7 s.

### 2.12. Statistical Analysis

The data were analyzed by SPSS 18.0 statistical software. The measured data are expressed as the mean ± standard deviation (mean ± SD). The measurement data were tested using Student’s *t* test and a repeated-measurement analysis of variance (ANOVA). Assuming that the test level was determined by *α* = 0.05, *p* < 0.05 was considered statistically significant. The graph was drawn using GraphPad 5.0 software.

## 3. Results

### 3.1. Changes in Energy Metabolism in the Skeletal Muscle of Cachectic Mice

**Figure 1:**
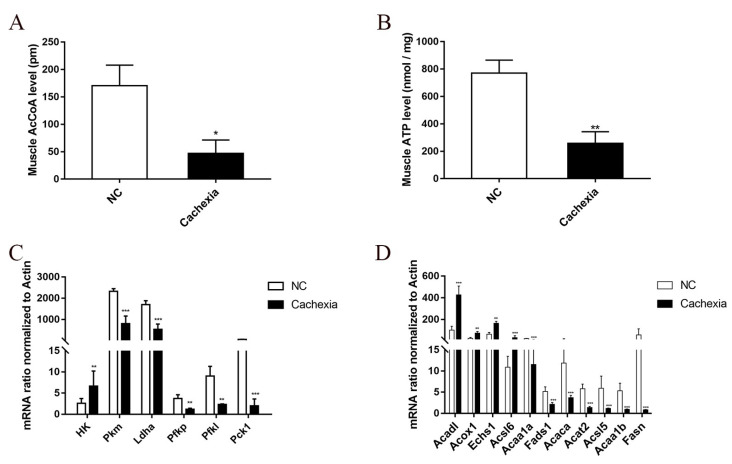
Alterations in skeletal muscle metabolites and gene expression in a cancer cachexic mouse model. (**A**) Total and cytosolic AcCoA levels in the skeletal muscle from normal mice (NC) group and C26 cancer cachexia mice group. (**B**) Total and cytosolic ATP levels in skeletal muscle from the NC group and the cachectic group. (**C**) Validation of the up- and downregulated DEGs in the enriched KEGG pathways involved in glycolysis/gluconeogenesis metabolism. These DEGs were assessed by qPCR. (**D**) Validation of the up- and downregulated DEGs in the enriched KEGG pathways involved in fatty acid metabolism. These DEGs were assessed via RT-qPCR. Data are expressed as the mean ± SD, * *p* < 0.05, ** *p* < 0.01, *** *p* < 0.001, *n* = 10.

Cachexia is a metabolic disorder characterized by skeletal muscle energy wasting. To check the energy metabolism imbalance, we first assessed the levels of acetyl-coenzyme A (AcCoA) in cachectic muscle, which is thought to be a common integrator of sugar, lipid, and protein catabolism in C26 tumor-bearing cachexia mice. The cachexia mice showed decreased levels of skeletal muscle AcCoA, as shown in Figure 1A. Meanwhile, the amount of adenosine triphosphate (ATP), the most important compound that provides energy to many processes in living cells, was markedly decreased in the cachexia group compared with the NC group, which was consistent with the AcCoA measurement (Figure 1B). In line with this result, we also measured the mRNA expression levels of the key rate-limiting enzymes involved in glucose and lipid metabolism, as shown in Figure 1C. In addition to the increased expression of hexokinase (HK), there was a significantly decreased expression of pyruvate kinase M1/2 (PKM), lactate dehydrogenase A (LDHA), phosphofructokinase, platelets (PFKP), phosphofructokinase, liver type (PFKL), and phosphoenolpyruvate carboxykinase 1 (PCK1), indicating decreased carbohydrate utilization in the muscle of cachexia mice. Regarding lipid metabolism, we noticed that the expression of enzymes regulating *β*-oxidation was significantly increased, including medium-chain acyl-CoA dehydrogenase (Acad1), acyl-CoA oxidase 1 (Acox1), enoyl-CoA hydratase, and short-chain 1 (Echs1) (Figure 1D). In comparison, the expression levels of enzymes regulating lipogenesis, including fatty acid desaturase 1 (Fads1), fatty acid synthase (Fasn) and acetyl-CoA carboxylase alpha (Acaca), were significantly decreased, suggesting enhanced fatty acid utilization but decreased fatty acid biosynthesis metabolism. Overall, this observation indicated that the energy metabolism balance was disrupted in cancer cachectic skeletal muscle.

### 3.2. In Vivo 2-DG Treatment Protects against Cachectic Weight Loss by Reversing Abnormal Glucose Metabolism

**Figure 2:**
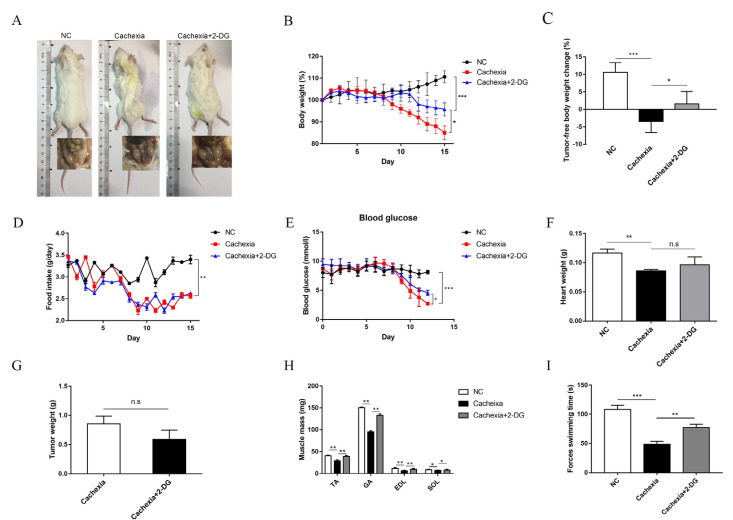
2-DG treatment alleviated cachexic-associated weight loss and muscle wasting in C26 tumor-bearing mice. (**A**) Representative general images and homologous groin images of the three groups. NC group: normal control mice, cachexia group: C26 tumor-bearing mice, cachexia+2-DG group: 2-DG-treated C26 tumor-bearing mice. (**B**) Body weight changes in the three groups (mean ± SD, *n* = 10). Day 0: tumor implantation. Day 7: 2-DG injection began. Day 15: terminal day. *** *p* < 0.001 denotes the NC group versus the cachexia group, * *p* < 0.05 denotes the cachexia group versus the cachexia+2-DG group. (**C**) Tumor removal weight changes in the three groups (mean ± SD, *n* = 10). *** *p* < 0.001 denotes the NC group versus the cachexia group, * *p* < 0.05 denotes the cachexia group versus the cachexia+2-DG group. (**D**) Daily food consumption in the three groups during the course of the experiment (mean ± SD, *n* = 10). ** *p* < 0.01 denotes the NC group versus the cachexia group. Neither group showed significant differences. (**E**) Blood glucose changes in the three groups (mean ± SD, *n* = 10). *** *p* < 0.001 denotes the NC group versus the cachexia group, * *p* < 0.05 denotes the cachexia group versus the cachexia+2-DG group. (**F**) The heart weight changes in the three groups (mean ± SD, *n* = 10). ** *p* < 0.01 denotes the NC group versus the cachexia group. No significant differences were found between the groups. (**G**) The tumor weight changes in the cachexia group and the cachexia+2-DG group (mean ± SD, *n* = 10). Neither group showed significant differences. (**H**) Skeletal muscle masses of tibialis anterior (TA), gastrocnemius (GA), extensor digitorum longus (EDL), and soleus (SOL). Data are shown as the mean ± SD, *n* = 10, ** *p* < 0.01, * *p* < 0.05. (**I**) Forced swimming time. *** *p* < 0.001 denotes the NC group versus the cachexia group, ** *p* < 0.01 denotes the cachexia group versus the cachexia+2-DG group. n.s. means not significant.

Glucose is the primary source of energy for cellular biosynthesis and generation. In line with the metabolic disorder of decreased AcCoA and ATP generation, we found a significantly elevated expression of hexokinase (HK) in skeletal muscle, which is the first rate-limiting step of glucose metabolism (Figure 1C). To analyze the contribution of HK overactivation to the development of metabolic disorders in cachectic muscle, 2-DG, a glucose analog that inhibits glycolysis by acting on HK, was used to block the HK function [28]. In the C26 adenocarcinoma-induced cachexia mouse model, administration of 2-DG in vivo significantly alleviated cachectic symptoms, including reduced body weight loss and increased lean mass, compared with the no-treatment cachectic mouse group, but preservation of inguinal white adipose tissue (iWAT) was not observed (Figure 2A,B). The tumor-free body weight change rate was significantly reduced (Figure 2C). Additionally, we noticed that the 2-DG treatment reversed tumor-induced weight loss independent of food intake (Figure 2D). In addition, we measured the blood glucose content in mice. Compared with the NC group, there was a significant reduction in tumor-bearing mice, and using 2-DG increased blood sugar levels (Figure 2E). To explore the influence of 2-DG on cardiac muscle and tumor growth, we measured the terminal weight of the heart and tumor. As the results showed, the cachexia group had a lower heart weight than the NC group, and the 2-DG treatment had no influence on the decreased heart weight (Figure 2F). Additionally, the 2-DG treatment had no significant influence on tumor growth (Figure 2G), precluding the possibility that the protective mechanism of 2-DG in preventing cachexia weight loss is due to its antitumor function. Additionally, we determined the muscle mass of the tibialis anterior (TA), gastrocnemius (GA), extensor digitorum longus (EDL), and soleus (SOL) in the three groups. We found that the 2-DG treatment protected skeletal muscle from atrophy in these four muscle groups (Figure 2H). To further explore changes in skeletal muscle function, we performed a forced swimming test, and the results showed that the 2-DG treatment markedly prolonged the swimming exhaustive time in tumor-bearing mice (Figure 2I).

### 3.3. 2-DG Treatment Prevents Cachectic Muscle Wasting by Inhibiting UPS and ALP Activation

**Figure 3:**
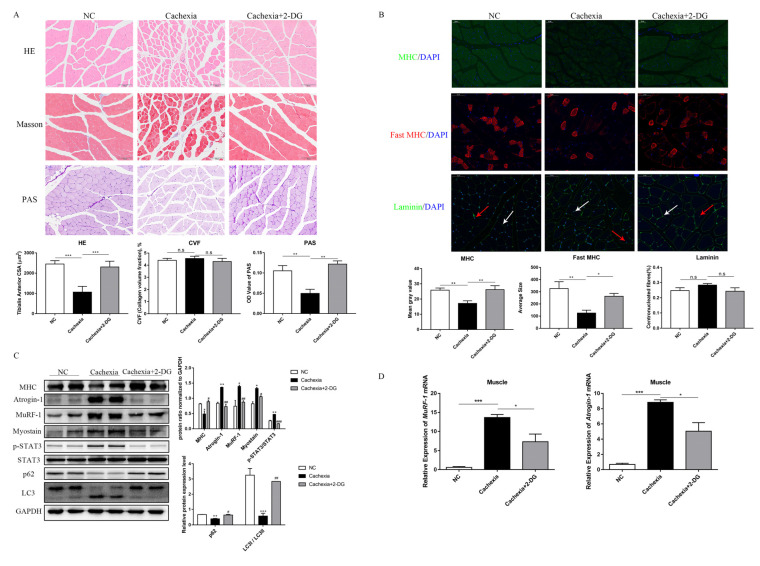
2-DG protects against cachexic-associated muscle wasting by inhibiting USP and ALP pathway activation. (**A**) Images showing the morphological changes in the muscles of the three groups using H&E staining. Scale bar: 100 µm. Average size of muscle fiber cross-sectional area in the three groups. Representative Masson’s trichrome staining of paraffin sections from muscle fibers of different groups of mice is shown. Scale bar: 100 µm. Using ImageJ, the collagen volume fraction (CVF) was plotted in a bar graph. The glycogen content was determined by periodic acid–Schiff (PAS) staining. Scale bar: 50 µm. In the histogram, the optical density (OD) of PAS staining is shown. (**B**) Gastrocnemius myosin heavy-chain (MHC) expression was examined by immunofluorescence staining. Scale bar: 50 µm. MHC, green; DAPI, blue. The bar graph shows the mean gray value of different groups. Antibodies against the fast myosin heavy-chain (MyHC) were used to stain gastrocnemius sections. Nuclei were detected using DAPI. Laminin and DAPI antibodies were used to stain gastrocnemius sections. Centrally nucleated fibers are indicated by red arrows. DAPI-labeled nuclei are indicated by white arrows. In the histogram, the number of centrally nucleated fibers is expressed as a percentage of the total number of fibers. (**C**) Western blot evaluated the MHC, atrogin-1, MuRF-1, myostatin, p-STAT3, STAT3, p62, and LC3 expression in the three groups (mean ± SD, *n* = 10). * *p* < 0.05, ** *p* < 0.01, *** *p* < 0.001 denotes the NC group versus the cachexia group; # *p* < 0.05, ## *p* < 0.01, ### *p* < 0.001 denotes the cachexia group versus the cachexia+2-DG group. (**D**) Total RNA was extracted from the gastrocnemius of the three groups, and the expression levels of atrogin-1 and MuRF-1 were evaluated using RT-PCR. Data are expressed as the mean ± SD, * *p* < 0.05, *** *p* < 0.001, *n* = 10. n.s. means not significant.

H&E staining and Masson’s trichrome staining were used to investigate the protective effect of 2-DG on skeletal muscle wasting. Histologically, the protective effect of 2-DG was demonstrated by an increase in myofiber size compared with the cachexia group (Figure 3A). Masson’s trichrome staining of mouse skeletal muscle paraffin sections revealed few differences in collagen deposition among the three groups (Figure 3A). As an important substrate of skeletal muscle energy metabolism, glycogen was decreased in cachexic mice and the 2-DG treatment increased muscular glycogen in cachexic mice. (Figure 3A). The type shift of muscle fibers is closely related to muscle function. Immunofluorescence staining of the myosin heavy-chain (MHC) showed that 2-DG increased the number of fast-twitch fibers (Figure 3B). The presence of centrally located nuclei in fibers suggests that degeneration–regeneration processes are not involved in muscle wasting caused by cachexia, and that 2-DG has no effect on this process (Figure 3B). Immunofluorescence and Western blot assays both showed MHC (MyHC, myosin heavy-chain) loss in C26 tumor-bearing mice when compared to the NC group, and the 2-DG treatment restored its expression (Figure 3B,C). The activation of STAT3 and expression of atrophy markers were then examined in mouse muscles. To regulate skeletal muscle atrophy, muscle RING finger-1 (MuRF-1) and muscle atrophy F-box (MAFbx, also known as atrogin-1) are essential ubiquitin ligase enzymes (E3) [30]. Our results demonstrated that the protein levels of atrogin-1, MuRF-1, myostatin, and *p*-STAT3 were higher in the cachexia group than in the NC group. The expression of atrogin-1, MuRF-1, myostatin, and *p*-STAT3 was significantly reduced by the 2-DG treatment (Figure 3C) and prevented the transcriptional activation of MuRF-1 and atrogin-1, which was markedly increased in the cachexia group (Figure 3D). These findings suggest that the 2-DG treatment could inhibit ubiquitination pathway degradation in the skeletal muscle of C26 tumor-bearing mice. Furthermore, to confirm whether autophagy activation was involved in cachexia, we performed Western blot assays to detect LC3 and p62. In tumor-bearing mice, we found a shift in the expression of LC-I to LC3-II, as well as an increase in p62 protein expression, indicating increased ALP activity (Figure 3C), and administration of 2-DG attenuated this increased ALP activity (Figure 3C). These changes in ALP activity established that ALP played a critical role in cancer-induced cachexia and that the 2-DG treatment was capable of inhibiting autophagy.

### 3.4. 2-DG Treatment Promotes Ketogenesis in the Liver and Enhances Ketone Utilization in Cachectic Skeletal Muscle

**Figure 4:**
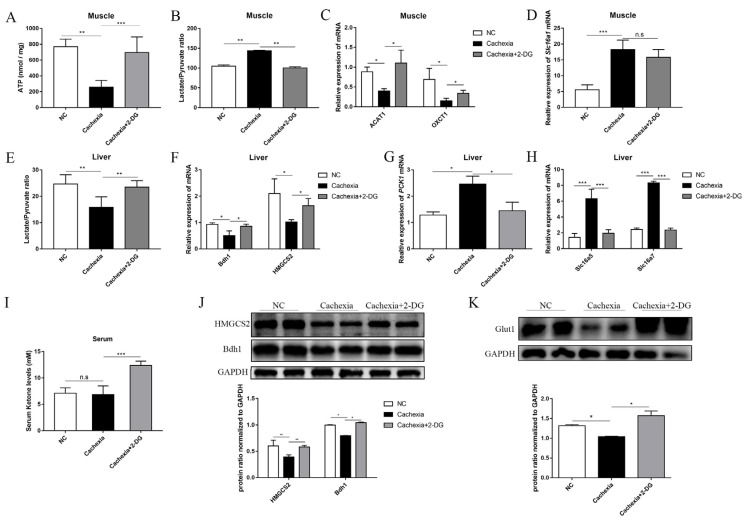
2-DG treatment increases ketogenesis in the liver and promotes ketone body utilization in skeletal muscle in C26 tumor-bearing mice. (**A**) The measurement of total and cytosolic ATP levels in the skeletal muscle from the NC group, cachexia group, and cachexia+2-DG group. (**B**) Ratio of lactate to pyruvate in the skeletal muscle from the NC group, cachexia group, and cachexia+2-DG group. (**C**) Total RNA was extracted from the gastrocnemius of the three groups, and the expression levels of ACAT1 and OXCT1 in the gastrocnemius were measured via RT-PCR. (**D**) Total RNA was extracted from the gastrocnemius of the three groups, and the expression levels of Slc16a1 in the gastrocnemius were measured via RT-PCR. (**E**) Ratio of lactate to pyruvate in the liver from the NC group, cachexia group, and cachexia+2-DG group. (**F**,**G**) Total RNA was extracted from the livers of the three groups, and the expression levels of Bdh1, HMGCS2 (**F**), and PCK1 (**G**) in the livers were measured via RT-PCR. (**H**) Total RNA was extracted from the gastrocnemius of the three groups, and the expression levels of Slc16a5 and Slc16a7 in the gastrocnemius were measured via RT-PCR. (**I**) Measurement of serum ketone levels in the skeletal muscle from the NC group, cachexia group, and cachexia+2-DG group. (**J**,**K**) Western blot analysis was used to evaluate HMGCS2 and Bdh1 expression in the livers of the three groups. Data are expressed as the mean ± SD, * *p* < 0.05, ** *p* < 0.01, *** *p* < 0.001, *n* = 10. n.s. means not significant.

We next measured the amount of ATP generation in these groups using a luminometer to compare the energy equivalents. In skeletal muscle, the cachexia group produced significantly less ATP than the NC group, indicating that energy production was restricted in cachectic mice. Notably, cachexia plus 2-DG treatment restored ATP generation compared with that in the untreated cachexia group (Figure 4A). We next determined the muscle lactate and pyruvate contents in mice to assess the function of 2-DG in inhibiting glycolysis. We observed that the muscle lactate/pyruvate ratio of the cachexia group was significantly higher than that of the NC group, which further supports the notion that cachectic muscle manifests an altered glucose metabolic pattern by switching oxidative phosphorylation into glycolysis, and the increased lactate/pyruvate ratio was reversed in response to 2-DG treatment (Figure 4B). Interestingly, we observed the opposite result in liver tissue (Figure 4D), which might be due to increased gluconeogenesis. Apart from this, we identified the protein level of Glut1, a major glucose transporter in mammals. The level of Glut1 was markedly decreased in the cachexia mice compared with the NC group, and using 2-DG reversed this change (Figure 4K). 2-DG exerts a compensatory effect on the liver by increasing alternative substrates, primarily ketone bodies, which serve as alternative energy substrates for the heart, muscles, and brain [31]. The serum level of ketone bodies was examined to determine whether 2-DG could promote ketogenesis in the mouse model. 2-DG significantly increased serum ketone bodies, whereas the NC and cachexia groups did not exhibit significant differences (Figure 4I). When fatty acids are oxidized in the liver, ketones are produced as a byproduct [32]. As a result, we determined the mRNA expression levels of 3-hydroxybutyrate dehydrogenase 1 (Bdh1) and 3-hydroxy-3-methylglutaryl-CoA synthase 2 (HMGCS2), the two critical rate-limiting enzymes involved in liver ketogenesis. Bdh1 and HMGCS2 mRNA and protein expression levels were significantly higher in the 2-DG-treated cachexia group than in the untreated cachexia model group, indicating that ketogenesis was activated in response to the 2-DG treatment (Figure 4F). Furthermore, the PCK1 mRNA level was higher in the cachexia group than in the NC group, and the application of 2-DG reversed this change (Figure 4G). Additionally, the expression of two critical mitochondrial enzymes, succinyl-CoA: 3-ketoacid CoA transferase (OXCT1) and acetyl-CoA acetyltransferase 1 (ACAT1), which convert the ketone bodies to acetyl-CoA, was significantly increased in response to the 2-DG treatment, as assessed via RT-PCR (Figure 4C). Furthermore, we detected the mRNA expression of lactate transporters Slc16a1 (in muscle), Slc16a5, and Slc16a7 (in liver) and found that the levels of all three lactate transporters were upregulated in the cachexia group compared with the NC group, and the 2-DG treatment reversed this change (Figure 4D,H). These findings indicate that the 2-DG treatment could facilitate ketogenesis and ketone body utilization to meet energy demands.

### 3.5. 2-DG Treatment Prevents Myotube Atrophy by Inhibiting Ubiquitination Degradation and Autophagy in a C26 Conditional Medium-Induced Muscle Atrophy Cell Model

**Figure 5:**
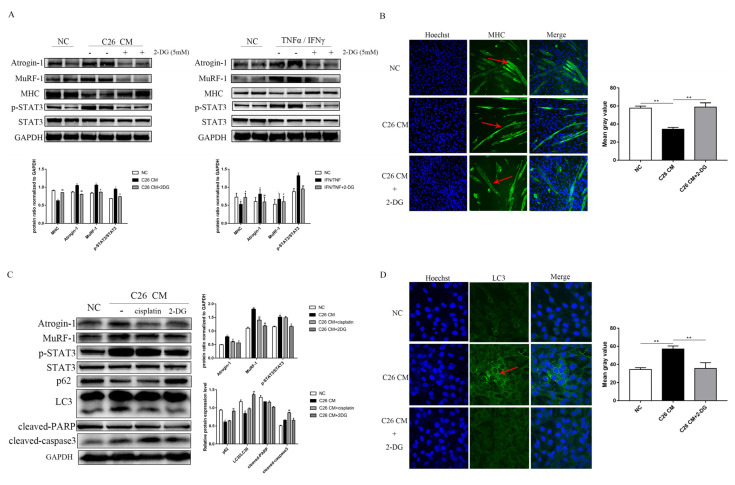
Supplementation with 2-DG reduces cachexic muscle atrophy by blocking USP and ALP pathway activation in C26 conditioned medium-treated C2C12 myotubes. (**A**) Western blot analysis was used to evaluate atrogin-1, MuRF-1, MHC, p-STAT3, and STAT3 expression in three groups between two cachexia cell lines models. (**B**) Myosin heavy-chain (MHC) expression in the cell model was evaluated by immunofluorescence staining. Scale bar: 50 µM. MHC, green; Hoechst, blue. The bar graph shows the mean gray value of different groups. (**C**) Western blot analysis was used to evaluate atrogin-1, MuRF-1, p-STAT3, STAT3, p62, LC3, cleaved PARP, and cleaved caspase3 expression in the cell model of the four groups. Cells were treated with 5 mM 2-DG, 20 µM cisplatin, or 25 µM chloroquine for 24 h. (**D**) LC3 expression in the cell model was evaluated by immunofluorescence staining. Scale bar: 50 µM. LC3, green; Hoechst, blue. The bar graph shows the mean gray value of different groups. Data are expressed as the mean ± SD, * *p* < 0.05, ** *p* < 0.01 compared with NC groups; # *p* < 0.05, ## *p* < 0.01 compared with C26 CM group or TNF*α*/IFN*γ* group, *n* = 10.

To address the molecular mechanism of 2-DG on skeletal muscle wasting protection in vitro, we used two well-characterized cell line models of muscle atrophy, consisting of myotube cultures exposed to TNF*α*/IFN*γ* or using C26 conditioned media (C26 CM). STAT3 activation has been shown to be a critical transcription factor that is causally linked to cachexia phenotypes such as skeletal muscle wasting, cardiac dysfunction, and hypothalamic inflammation [33]. MuRF-1 and atrogin-1 protein levels were upregulated in two cachexia cell line models, which was accompanied by increased STAT3 pathway activation and MHC downregulation. Notably, the 2-DG treatment restored MHC expression (Figure 5A,B) while inhibiting STAT3 activation and protein proteasome degradation-associated molecular markers, including MuRF1 and atrogin-1, in C2C12 myotubes (Figure 5A). Immunofluorescence of MHC showed that the administration of 2-DG prevented the decrease in the myotube size, myotube diameter, and myotube area (red arrow) induced by C26 CM (Figure 5B). The C26 CM treatment resulted in the degradation of C2C12 myotubes, whereas the 2-DG treatment resulted in the significant protection of myotubes against C26 CM-induced atrophy (Figure 5C). At the same time, we examined the expression of p62, which was used as an autophagy marker via Western blotting, and LC3, which changed from LC3-I to LC3-II when autophagy was activated. Compared to the C26 CM group, the 2-DG treatment reduced p62 accumulation and the LC3-II/LC3-I ratio, indicating the inhibitory effect of 2-DG against cachexia-associated muscle ALP activation. Furthermore, we also confirmed that the altered protein expression mentioned was not caused by apoptosis, since the apoptosis inducer compound cisplatin failed to induce this effect (Figure 5C). Autophagy flux was tested in experiments with fluorescence labeling to monitor autophagy activation in 2-DG-treated C2C12 myotubes. Increased autophagic vesicles (red arrow) accumulated in C26 CM-treated C2C12 myotubes, but autophagic flux was significantly reduced after 24 h of 2-DG treatment (Figure 5D). These findings suggest that 2-DG could inhibit the ubiquitin–proteasome system and autophagic–lysosomal pathway activation in vitro.

### 3.6. 2-DG Treatment Promotes the Utilization of Ketones and Alters Energy Metabolism in a Muscle Atrophy Cell Model

**Figure 6:**
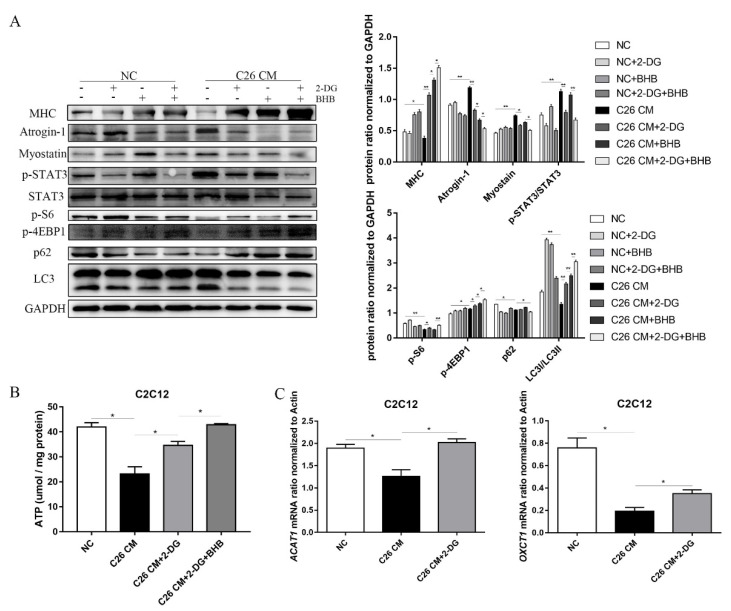
The combination of 2-DG and BHB treatment further reduces cachexic muscle wasting in C2C12 myotubes treated with C26 conditioned medium. (**A**) Western blot analysis was used to evaluate MHC, atrogin-1, myostatin, p-STAT3, STAT3, p-S6, p-4EBP1, p62, and LC3 expression in different groups. (**B**) Measurement of total and cytosolic ATP levels in C2C12 cells from the NC group, C26 CM group, C26 CM+2-DG group, and C26 CM+2-DG+BHB group. (**C**) Total RNA was extracted from the C2C12 cells of the three groups, and the expression levels of Bdh1 and HMGCS2 in C2C12 cells were measured via RT-PCR. Data are expressed as the mean ± SD, * *p* < 0.05, ** *p* < 0.01, *n* = 10.

To determine whether ketone bodies were involved in the protective effects of 2-DG on myotube atrophy, we supplemented the culture medium with *β*-hydroxybutyrate (BHB), the most abundant ketone body in mammals. We observed that the combined treatment of 2-DG and BHB resulted in a more significant inhibition of UPP-associated protein expression, including atrogin-1, MuRF-1, and myostatin. STAT3 signaling pathway activation was also significantly blocked, and protein-related autophagy was also significantly inhibited (Figure 6A), which was accompanied by increased mTOR signaling pathway activation in C2C12 myotubes in response to the 2-DG and BHB combined treatment. The alterations of these proteins revealed that using BHB combined with 2-DG had a significantly enhanced protective effect against C26 CM-induced myotube degradation compared with using 2-DG alone (Figure 6A). Notably, the production of ATP was greater when BHB was combined with 2-DG compared with the 2-DG treatment alone (Figure 6B). Additionally, we determined the levels of ACAT1 and OXCT1 mRNA expression in response to the 2-DG treatment. We found that the expression levels of ACAT1 and OXCT1 were significantly increased in the 2-DG treatment group compared to the C26 CM group (Figure 6C), which corresponded with our in vivo results. Overall, our findings suggest that the 2-DG treatment could prevent C2C12 myotube atrophy by increasing ketone body utilization and improving energy metabolism.

## 4. Discussion

Cancer cachexia results from the imbalance between energy supply and energy expenditure; apart from the decreased energy supply caused by anorexia, several types of futile cycles, such as the Cori cycle, cause the higher energy expenditure [34]. Even in the presence of oxygen, the energy generated by oxidative phosphorylation in the mitochondria is insufficient for most cancer cells; thus, the energy generated by anaerobic glycolysis is increased to compensate (the Warburg effect) [21], which is less effective for energy generation. Since most cancer cells have an increased demand for glucose, to compensate for the supplementation of glucose and reduce the plasma level of lactate, increased hepatic glucose production by gluconeogenesis is commonly observed in cancer cachexia [35]. In our study, we observed that cachectic myotubes secreted more lactate than the control healthy myotubes. There was increased transcriptional expression of the glucose phosphorylation enzyme hexokinase (HK), the key enzyme for the first and rate-limiting step in the glycolytic pathway, which regulated glucose metabolism and was accompanied by decreased AcCoA and ATP generation, indicating that anaerobic glycolysis, rather than oxidative phosphorylation, is the major glucose utilization pattern in cachectic skeletal muscles. These results indicated that cachectic myotubes exhibit a similar metabolic pattern to cancer cells (known as the Warburg effect) under aerobic conditions, which produces lactate [36]. Lactate produced by tumor cells and cachectic muscles is transported to the liver and converted to glucose via the Cori cycle, since the liver requires energy to carry out gluconeogenesis, and anaerobic glycolysis is an energy-inefficient process. The overwhelming activation of the Cori cycle is, therefore, a futile cycle and energy-wasting process [37]. In addition to altered glucose utilization and increased aerobic glycolysis, cancer patients have also been shown to have glucose intolerance and the elevated production of hepatic glucose [35]. Increased hepatic glucose production may be stimulated by an increased glucose requirement, which might explain why, apart from the increased energy expenditure, elevated plasma glucose-triggered insulin resistance and excess catabolism are also common pathological features of cancer cachexia. Additionally, the enhanced generation of lactate by aerobic glycolysis has been found to be linked to cancer metastases and recurrence [38]. Ketogenic diets have been shown to be a very effective method for treating experimental cancer cachexia. In ketogenic diet therapy for cancer, the Warburg effect is targeted, as it limits the energy sources for cancer cells by limiting carbohydrates and provides healthy cells with fatty acids and ketone bodies [39,40,41]. Cachectic muscle retains the ability to metabolize ketone bodies, although most cancer cells lack the mitochondrial enzymes required for ketone body metabolism [42]. A cachexic mouse model of non-small-cell lung cancer (NSCLC) demonstrated a reduced liver fatty acid oxidation capacity and increased gluconeogenesis markers, which correlate with significantly reduced generation of ketone bodies in the liver and decreased serum ketone body levels [22]. Our study fully supports their finding because a significant decrease in serum ketone bodies was also observed in our C26 cachexic mouse model, and the expression of the enzymes controlling ketone synthesis, including Bdh1 and HMGCS2, was significantly inhibited. Based on the finding that 2-DG could revert the destroyed energy metabolism balance in cancer-induced cachexia animals, we speculated that the involved mechanism was, on the one hand, slowing down the Cori cycle by blocking aerobic glycolysis and lactate production in cachexic skeletal muscle; on the other hand, 2-DG administration in vivo was found to be able to increase ketone synthesis and utilization. Our data showed that the 2-DG treatment increased the supply of ketone bodies and enhanced the mRNA expression of Bdh1 and HMGCS2, the key rate-limiting enzymes involved in liver ketogenesis. A previous study indicated that ketone bodies can serve as auxiliary and alternative fuels during a biochemical crisis [43]. Another important finding in our study is that 2-DG treatment could increase ketone body utilization. For glucose metabolism, pyruvate dehydrogenase (PDH) catalyzes pyruvate decarboxylation, the first step in converting pyruvate to acetyl-CoA. Cachectic conditions have been reported to significantly decrease PDH activity [36]. To compensate for the deficits in PDH capacity, an alternative fuel that does not require PDH, such as ketones, can be used to generate acetyl-CoA for ATP synthesis. In our study, low levels of skeletal muscle AcCoA and ATP were observed in C26 tumor-bearing mice. Lactate content and glycolytic enzymes were significantly reduced by 2-DG treatment, which was accompanied by significantly increased AcCoA and ATP levels in the skeletal muscle of C26 tumor-bearing mice. The expression of ACAT1, an enzyme involved in ketone body utilization, was significantly upregulated. Synthetic glucose analog 2-DG is capable of entering cells and interfering with glycolysis and the production of ATP. Due to the structural similarities between 2-DG and glucose, it was hypothesized that 2-DG would act as a competitive inhibitor of glucose metabolism, and, therefore, could have a significant impact on the growth of glucose-dependent cells, such as cancer cells undergoing aerobic glycolysis. Thus, it was one of the first compounds to be studied for its ability to interfere with tumor metabolism [44]. In addition to inhibiting glycolysis through the formation and accumulation of 2-deoxy-d-glucose-6-phosphate (2-DG6P), 2-DG also induces cell death by inhibiting hexokinase and glucose-6-phosphate isomerase. 2-DG also inhibits other molecular processes in addition to glycolysis [45]. Apart from the role of 2-DG in glucose metabolism, we also explored its role in adipose tissue and found that 2-DG has no protective effect on inguinal fat in cachectic mice (Figure 2A). The UPS and ALP are two major proteolytic systems that degrade muscle proteins [46]. Studies have demonstrated that ubiquitin–proteasomal system activation is induced by various catabolic conditions through the upregulation of ubiquitin ligase expression [47]. A key step in muscle protein degradation by UPS is the activation of the E3 ubiquitin ligase and the subsequent increased protein ubiquitination. MURF1 and atrogin-1 are muscle-specific E3 ubiquitin ligases that play a key role in muscle loss and bind various muscle proteins, including troponin I, actin, and MHC. [48]. In our study, we observed the significantly increased expression of MuRF1 and atrogin-1, accompanied by the decreased expression of MHC, confirming the involvement of UPS activation in cachectic muscle wasting. Importantly, our findings also confirmed that the 2-DG treatment successfully suppressed the expression of both MuRF-1 and atrogin-1 and recovered the MHC expression. The prolonged activation of the STAT3 pathway could stimulate UPS activity and contribute to cachectic muscle wasting, which has been reported by our group and others [49,50] and was also suppressed in response to 2-DG treatment, both in vivo and in vitro. ALP is an additional pathway for the induction of cachectic muscle wasting, which is responsible for the degradation of muscle-specific cellular organelle proteins. In this process, the reduced amounts of p62 and LC3 are involved in autophagosome formation [46]. In our study, we detected a significant decrease in p62 and an increase in LC3 II/I ratio protein levels in cachectic mice and cell models. The 2-DG treatment markedly increased p62 accumulation and decreased the LC3 II/I ratio in both in vivo and in vitro cachectic models. Due to increased autophagic sequestration or decreased autophagosome clearance, LC3 II/I accumulation can occur during autophagy. We therefore conducted a flux experiment to monitor autophagy activation, and the results showed that LC3 II/I levels increased more in myotubes, indicating that the LC3 II/I ratio increased due to increased autophagic activity, and 2-DG treatment attenuated this effect.

The ATP level and activation of the AMPK pathway have been found to regulate autophagy activation. Our results confirmed this point, since the enhanced activation of the ALP in cachectic muscle correlated with the reduced generation of ATP and decreased levels of AcCoA, whereas in response to the 2-DG treatment, the increased generation of ATP and AcCoA correlated with the decreased activation of ALP, indicating that intercellular energy exhaustion is likely the major cause for the development of cachectic muscle wasting. To further verify the protective effect of 2-DG and its correlation with ketone body utilization, the activation of the UPS, and autophagy, we added *β*-hydroxybutyrate (BHB), which is the most abundant ketone body in mammals, to the culture medium in the in vitro cachectic muscle wasting cell model. The results showed enhanced protection of cachectic muscle wasting and significantly reduced activation of the UPS and ALP signaling pathways in the 2-DG plus BHB group compared with the 2-DG treatment alone. These findings support the notion that inhibiting abnormal glucose metabolism in cachectic muscle can change its metabolic pattern and that increasing the generation and utilization of ketone bodies, which can restore intracellular energy supplementation, is an important approach for the treatment of muscle wasting in cancer cachexia.

However, there are some limitations to our study. First, for steady-state conditions, single-timepoint labeling experiments may be insufficient. However, based on the real-time ECAR and OCR measurements, a solid plateau was observed at the time of these measurements, indicating that cells were approaching steady-state conditions. Second, we only focused on the effect of 2-DG on glycolysis in cachexia, and did not explore the interaction of 2-DG with the pentose phosphate pathway (PPP) or the interaction of 2-DG with protein glycosylation in cachexia. These are worthy of further research in the future.

## 5. Conclusions

**Figure 7:**
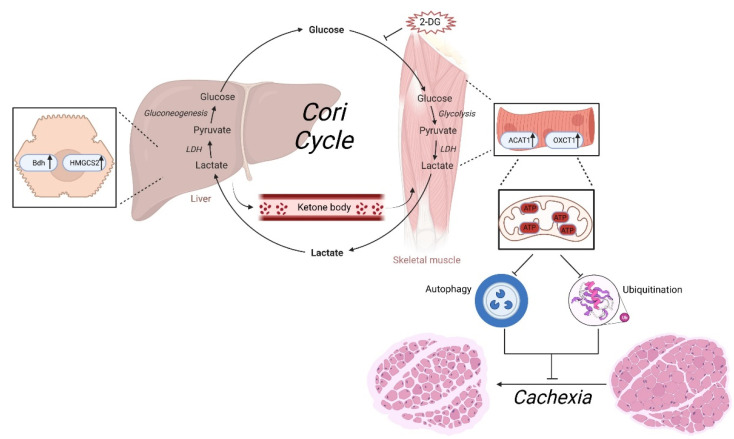
Graphical summary of the mechanism by which 2-DG ameliorates the cachexia phenotype by altering ketone body metabolism.

In the current study, we provided evidence that strategies targeting hexokinase (HK) using a 2-DG treatment attenuated cachexia-associated skeletal muscle wasting in a C26 tumor-bearing mouse model. These findings have translational significance, as they demonstrate that administration of 2-DG not only blocks the futile Cori cycle, but also promotes the ketone metabolic pathway, thus restoring ATP generation, which subsequently inhibits the activation of the UPS and ALP pathways, protecting against cachexia-associated muscle atrophy (Figure 7). Further clinical studies are needed to confirm the protective role of 2-DG in muscle wasting and prolong the survival time in clinical cancer cachexia patients. Our results could have important implications for reforming drug prescriptions in the clinical management of cancer cachexia.

## Data Availability

The datasets generated and analyzed during the current study are available from the corresponding author on reasonable request.

## Supplementary Materials

The following supporting information can be downloaded at: https://www.mdpi.com/article/10.3390/cells11192987/s1, Table S1: Detailed primer sequences are provided.
