# Supplementary material for: 2-Deoxy-D-glucose Alleviates Cancer Cachexia-Induced Muscle Wasting by Enhancing Ketone Metabolism and Inhibiting the Cori Cycle
Source: Cells. 2022 Sep 25;11(19):2987. doi: 10.3390/cells11192987 (PMC9562633; doi:10.3390/cells11192987)
Supplement: Supplementary file 1 [file cells-11-02987-s001.zip › Supplementary Table S1.pdf]

Table S1. Sequence of primers used in qRT-PCR

Gene	Forward	Reverse
MuRF-1	5'-ACCTGCTGGTGGAAAACATC-3'	5'-AGGAGCAAGTAGGCACCTCA-3'
Atrogin-1	5'-ATTCTACACTGGCAGCAGCA-3'	5'-TCAGCCTCTGCATGATGTTC-3'
HMGCS2	5'-TGGTTCAAGACAGGGACACAGAAC-3'	5'-AGAGGAATACCAGGGCCCAACAAT-3'
Bdh1	5'-TGCAACAGTGAAGAGGTGGAGAAG-3'	5'-CAAACGTTGAGATGCCTGCGTTGT-3'
ACAT1	5'-GCAGGGAAGTTTGCCAGTGAGA-3'	5'-GAACACGGTCTTGAGCTTTGGC-3'
HK	5'-ACCAAGCGGTATCAGCATGTG-3'	5'-TGGACTTCTCTGTGATTGGCA-3'
Pkm	5'-GTACCCCCATCCCAGTTCCA-3'	5'-TCTGAGGTCTCACACAGGAAA-3'
Ldha	5'-GGCTACAAGCATCTTGAGAGC-3'	5'-TGCTGGATAAGTTCACCCTTCA-3'
Pfkp	5'-CGCCTATCCGAAGTACCTGGA-3'	5'-CCCCGTGTAGATTCCCATGC-3'
Pfkl	5'-GGAGGCGAGAACATCAAGCC-3'	5'-GCACTGCCAATAATGGTGCC-3'
Pck1	5'-CTGCATAACGGTCTGGACTTC-3'	5'-GCCTTCCACGAACTTCCTCAC-3'
Acadl	5'-TTTCCTCGGAGCATGACATTTT-3'	5'-GCCAGCTTTTTCCCAGACCT-3'
Acox1	5'-CCGCCACCTTCAATCCAGAG-3'	5'-CAAGTTCTCGATTTCTCGACGG-3'
Echs1	5'-CCCAGAACTACGGCGCTTC-3'	5'-CCACGCTGCTATTCTTTCCTT-3'
Acsl6	5'-AAGTGACAGAGAGTCAGTGGG-3'	5'-TAGGGCGGAGAGCCTTCAT-3'
Acaa1a	5'-TCTCCAGGACGTGAGGCTAAA-3'	5'-CGCTCAGAAATTGGGCGATG-3'
Fads1	5'-AGCACATGCCATACAACCATC-3'	5'-TTTCCGCTGAACCACAAAATAGA-3'
Acaca	5'-GATGAACCATCTCCGTTGGC-3'	5'-GACCCAATTATGAATCGGGAGTG-3'
Acat2	5'-CCCGTGGTCATCGTCTCAG-3'	5'-GGACAGGGCACCATTGAAGG-3'
Acsl5	5'-TCCTGACGTTTGGAACGGC-3'	5'-CTCCCTCAATCCCCACAGAC-3'
Acaa1b	5'-CAGGACGTGAAGCTAAAGCCT-3'	5'-CTCCGAAGTTATCCCCATAGGAA-3'
Fasn	5'-GGAGGTGGTGATAGCCGGTAT-3'	5'-TGGGTAATCCATAGAGCCCAG-3'
Slc16a1	5'-GGTGGGCAGTGTTAGTCGG-3'	5'-GATAGGACCTCCAGCATACATGA-3'
Slc16a5	5'-CACCTGCATCGGTGTCTTCTT-3'	5'-AAGGAAACCACGAGGTCTCAC-3'
Slc16a7	5'-GGGCTGGGTCGTAGTCTGT-3'	5'-ATCCAAGCGATCTGACTGGAG-3'
Actin	5'-CTAAGGCCAACCGTGAAAAG-3'	5'-ACCAGAGGCATACAGGGACA-3'
